# The Amphibious Mudskipper: A Unique Model Bridging the Gap of Central Actions of Osmoregulatory Hormones Between Terrestrial and Aquatic Vertebrates

**DOI:** 10.3389/fphys.2018.01112

**Published:** 2018-08-14

**Authors:** Yukitoshi Katayama, Tatsuya Sakamoto, Keiko Takanami, Yoshio Takei

**Affiliations:** ^1^Physiology Section, Atmosphere and Ocean Research Institute, The University of Tokyo, Kashiwa, Japan; ^2^Ushimado Marine Institute, Faculty of Science, Okayama University, Setouchi, Japan; ^3^Mouse Genomics Resource Laboratory, National Institute of Genetics, Mishima, Japan

**Keywords:** amphibious behavior, osmoregulation, angiotensin II, neurohypophysial hormones, corticosteroids, thirst, social behavior, mudskipper

## Abstract

Body fluid regulation, or osmoregulation, continues to be a major topic in comparative physiology, and teleost fishes have been the subject of intensive research. Great progress has been made in understanding the osmoregulatory mechanisms including drinking behavior in teleosts and mammals. Mudskipper gobies can bridge the gap from aquatic to terrestrial habitats by their amphibious behavior, but the studies are yet emerging. In this review, we introduce this unique teleost as a model to study osmoregulatory behaviors, particularly amphibious behaviors regulated by the central action of hormones. Regarding drinking behavior of mammals, a thirst sensation is aroused by angiotensin II (Ang II) through direct actions on the forebrain circumventricular structures, which predominantly motivates them to search for water and take it into the mouth for drinking. By contrast, aquatic teleosts can drink water that is constantly present in their mouth only by reflex swallowing, and Ang II induces swallowing by acting on the hindbrain circumventricular organ without inducing thirst. In mudskippers, however, through the loss of buccal water by swallowing, which appears to induce buccal drying on land, Ang II motivates these fishes to move to water for drinking. Thus, mudskippers revealed a unique thirst regulation by sensory detection in the buccal cavity. In addition, the neurohypophysial hormones, isotocin (IT) and vasotocin (VT), promote migration to water via IT receptors in mudskippers. VT is also dipsogenic and the neurons in the forebrain may mediate their thirst. VT regulates social behaviors as well as osmoregulation. The VT-induced migration appears to be a submissive response of subordinate mudskippers to escape from competitive and dehydrating land. Together with implications of VT in aggression, mudskippers may bridge the multiple functions of neurohypophysial hormones. Interestingly, cortisol, an important hormone for seawater adaptation and stress response in teleosts, also stimulates the migration toward water, mediated possibly via the mineralocorticoid receptor. The corticosteroid system that is responsive to external stressors can accelerate emergence of migration to alternative habitats. In this review, we suggest this unique teleost as an important model to deepen insights into the behavioral roles of these hormones in relation to osmoregulation.

## Evolution of Body Fluid Regulation From Fishes to Tetrapods

Ionic concentration, osmolality, and volume of body fluids are important internal parameters that are tightly controlled in vertebrates by the ingestion and excretion of water and ions (Bourque, 2008). As vertebrates expanded their habitats from aquatic to terrestrial environments, terrestrial adaptation requires critical changes in the osmoregulatory and cardiovascular systems to counter both dehydration and gravity (Leow, 2015). To cope with dehydration, they drink water and reduce evaporative water loss from the body surface by a developed body integument consisting of layers of keratinized skin cells. In addition, the kidney of endothermic mammals and birds is equipped with juxtamedullary nephrons that can produce hyperosmotic urine, which is an adaptation to reduce water loss from excretion.

**FIGURE 1:**
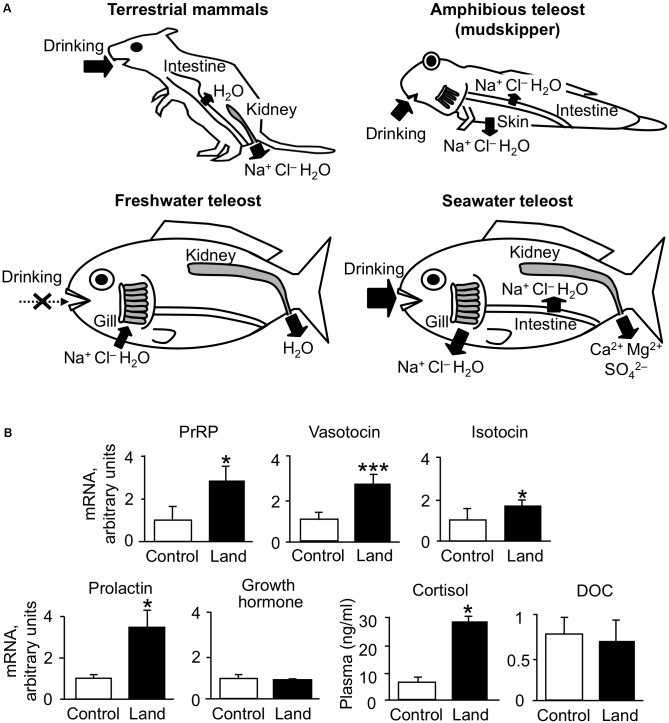
Environmental adaptations in vertebrates. **(A)** Osmoregulatory mechanisms in mammals and teleosts. Arrows show active and passive transport of ions and/or water. The osmoregulatory mechanisms are flexible in euryhaline species such as catadromous eels and anadromous salmonids, which switch ion and water regulation to opposite directions via active transport. In addition to aquatic teleosts, the amphibious and euryhaline mudskipper, which invades land in its lifecycle, is used for the study of osmoregulation. **(B)** Dynamics of osmoregulatory hormones in terrestrial adaptation of mudskippers. Cortisol and DOC are shown as plasma concentrations (Sakamoto et al., 2002, 2011), and the other hormones are shown as the expression of their genes in the brain of mudskippers in controls (in one-third seawater) or on land (*n* = 4–8) (Sakamoto et al., 2005a, 2015). Data are shown as the means ± SE. ^∗^*p* < 0.05, ^∗∗∗^*p* < 0.001 with *t*-test or Mann–Whitney *U*-test. PrRP, prolactin-releasing peptide; DOC, 11-deoxycorticosterone.

Similar to terrestrial tetrapods, teleost fishes are osmotic and ionic regulators and the ionic composition of their body fluis is similar to those of tetrapods, whose plasma osmolality is approximately one third of seawater regardless of the salinity that they inhabit (Evans, 2008). Their osmoregulatory ability might have allowed them to flourish in a wide range of aquatic environments including freshwater, seawater, and in particular cases allowed survival and success even on land (Takei, 2015). Marine teleosts exposed to severe dehydration drink seawater to cope with this problem (Hirano et al., 1972; Kobayashi et al., 1983; Perrott et al., 1992; Takei, 2015). After drinking, seawater is desalinated in the esophagus, and then water is absorbed together with NaCl in the intestine after isotonic dilution (Parmelee and Renfro, 1983; Nagashima and Ando, 1994; Takei et al., 2016). High amount of HCO_3_^-^ is secreted into intestinal luminal fluid so that Ca^2+^ and Mg^2+^ are removed by precipitation in the form of carbonate aggregates (Wilson et al., 2002; Kurita et al., 2008; Grosell, 2011). The excess monovalent ions such as Na^+^ and Cl^-^ are excreted from the branchial or cutaneous ionocytes (Uchida et al., 1996; Sakamoto et al., 2000; Seo et al., 2015) and divalent ions such as Ca^2+^, Mg^2+^, and SO_4_^2-^ are excreted from the kidney (Watanabe and Takei, 2011). In freshwater teleosts, uptake of environmental ions through the gill is activated for hyperosmoregulation (Takei et al., 2014). This action is mediated by ion transporters such as Na^+^-K^+^-ATPase (NKA) and Ca^2+^-ATPase (Hoenderop et al., 2005; Hwang et al., 2011). In these studies, species differences in osmoregulatory mechanisms and hormonal function have been found (Takei et al., 2014). Further, the osmoregulatory mechanisms are flexible in euryhaline or migratory species such as eels and salmonids, which experience drastic salinity changes during their life cycle and have to switch ion and water regulation to opposite directions via active transport (**Figure 1A**). Studies on these teleosts have highlighted pivotal roles of various hormones in adaptation to fluctuating environmental salinities (McCormick, 2001; Takei and McCormick, 2012).

Ample studies have clarified functions of osmoregulatory and cardiovascular hormones in terrestrial tetrapods and aquatic teleosts (McCormick and Bradshaw, 2006; Bourque, 2008; Mével et al., 2012; Takei et al., 2014; Leow, 2015). In teleosts, however, considerably less research effort is directed at determining their role in behaviors. In addition, it is little known how their functions are conserved or have evolved among diverse taxa through evolutionary time. An exception is drinking behavior induced by angiotensin II (Ang II). In mammals, circulating Ang II is a major factor in the increased thirst and sodium appetite of hypovolemia (Fitzsimons, 1998). These effects play important roles in sustaining the blood volume and blood pressure and would certainly have been evolutionarily advantageous. With regard to thirst, Ang II act on the thirst center to motivate terrestrial mammals to seek for and ingest water. Ingestion of water rapidly satiates thirst sensation by sensory detection of water in the gastrointestinal tract (Zimmerman et al., 2016). Ang II also acts in concert with vasopressin (VP) to decrease the loss of water (Fitzsimons, 1998). In aquatic teleosts, Ang II and neurohypophysial hormones similarly regulate drinking (Takei et al., 1979; Balment and Carrick, 1985; Perrott et al., 1992; Kozaka et al., 2003; Watanabe et al., 2007; Fuentes and Eddy, 2012). However, as we often found differences in the response to osmoregulatory hormones among teleost species (Kobayashi et al., 1983), a comparative approach may benefit deeper understanding on the function of osmoregulatory hormones, which will not be readily available when studying mammals exclusively.

## Amphibious Mudskipper as a Unique Model for Studying Osmoregulatory Behavior

Mudskipper fishes including *Periophthalmus modestus* are euryhaline species that can tolerate salinities ranging from 0 to 40 parts per thousand (ppt). They often experience rapid changes in salinity each day with tide in the estuary and so their osmoregulatory mechanisms are highly flexible. Furthermore, they spend the greater time of their lives out of water to feed and to escape from aquatic predators. They have acquired behavioral and physiological adaptations to amphibious lives (Clayton, 1993; Graham, 1997; Sakamoto and Ando, 2002; Sakamoto et al., 2005a). The roles of endocrine systems in their amphibious features have been investigated (**Table 1**). Because of the unique amphibious behavior (i.e., migration between terrestrial and aquatic areas), mudskippers may serve as a valuable experimental model to investigate the central actions of osmoregulatory hormones and to provide new insights into the evolution of hormonal actions during transition from aquatic to terrestrial lifestyle.

**Table 1:** Hormones involved in the amphibious habits of mudskippers.

Hormone	Dynamics under terrestrial condition	Effect on aquatic preference		Reference
		Treatment		
Vasotocin	+ (mRNA)	+ (IM^b^, ICV^c^)	via ITR^d^	Sakamoto et al., 2015
Isotocin	+ (mRNA)	+ (IM, ICV)	via ITR	Sakamoto et al., 2015
Prolactin-releasing peptide	+ (mRNA)	ND		Sakamoto et al., 2005a
Prolactin	+ (mRNA)	+ (IM)		Lee and Ip, 1987; Sakamoto et al., 2005a,b
3,5,3′-triiodo-_L_-thyronine	± (Plasma)	- (Immersion)		Lee and Ip, 1987; Sakamoto et al., 2011
Thyroxine	+ (Plasma)	ND		Lee and Ip, 1987
Cortisol	+ (Plasma)	+ (Immersion, ICV)	via GR^e^, MR^f^	Sakamoto et al., 2002, 2011
11-deoxycorticosterone	± (Plasma)	+ (Immersion, ICV)	via MR	Sakamoto et al., 2011
Angiotensin II	ND^a^	+ (IM, ICV)	for drinking	Katayama et al., 2018

*^a^ND: Not determined,^b^IM: Intramuscular injection, ^c^ICV: Intracerebroventricular injection,^d^ITR: Isotocin receptor, ^e^GR: Glucocorticoid receptor, ^f^MR: Mineralocorticoid receptor, +: Increase, -: Decrease, ± : No significant change.*

Which actions of osmoregulatory hormones have been conserved and/or exploited in this teleost? Among the accumulated data, we will focus on three topics in this review. First, we discuss the role of Ang II in drinking behavior. The drinking behavior of mudskippers is composed of migration to water, taking water into the mouth, and swallowing, which may most likely be associated with thirst. The second topic is the interaction between osmoregulation and social behavior, both of which are regulated by the neurohypophysial hormones, vasotocin (VT) and isotocin (IT). Finally, we introduce the role of corticosteroids in the amphibious behavior. Aldosterone is a major mineralocorticoid in mammals, but only minimally represented in teleosts. In teleosts, cortisol acts as mineralocorticoid as well as glucocorticoid (Takahashi and Sakamoto, 2013). Thus, cortisol action on the amphibious behavior has been investigated. We expect that this review will arouse further interest in the functional evolution of osmoregulatory hormones not only for fish endocrinologists but also for those working on other animals.

### Angiotensin II and Thirst-Motivated Migration

Comparative studies using various vertebrates such as teleosts, amphibians, and mammals suggest that adaptation to life on dry land with a full influence of the gravitational force necessitates an elaborate renin-angiotensin system to be evolved (Nishimura, 1978; Leow, 2015). In mammals, the renin-angiotensin cascade is initiated by the release of renin from the juxtaglomerular cells in the renal afferent arteriole. Renin is released by hypovolemia and subsequent decreases in perfusion pressure at the arteriole (Kobayashi and Takei, 1996; Nishimura, 2017). The principal action of the active principle, Ang II, is to restore blood volume by retaining NaCl and water. Ang II stimulates secretion of VP and aldosterone, thereby further contributing to volume retention. Indeed, loss of function of the renin-angiotensin or VP system resulted in a hypotensive phenotype (Doan et al., 2001; Fujiwara and Bichet, 2005). Since inhibitors of the renin-angiotensin system attenuate hypovolemia-induced drinking, plasma Ang II is closely related to extracellular dehydration (Kobayashi and Takei, 1996). Unlike in mammals, plasma Ang II levels increase by hyperosmotic stimulus (cellular dehydration) as well as by hypovolemic stimulus in teleosts (Nishimura, 1978; Tierney et al., 1995; Takei, 2000). Transfer from fresh water to seawater results in a small and transient increase in plasma Ang II concentration in parallel with plasma osmolality (Okawara et al., 1987). Thus, Ang II functions as a fast-acting hormone in response to fluctuation of environmental salinity (Takei et al., 2014). The dipsogenic effect of Ang II has been examined extensively in various vertebrates including teleost and elasmobranch fishes (Kobayashi et al., 1983; Perrott et al., 1992; Anderson et al., 2001; Fuentes and Eddy, 2012). Ang II is the most potent dipsogenic hormone thus far known in many vertebrate species (Fitzsimons, 1998; Takei, 2000; McKinley and Johnson, 2004).

**FIGURE 2:**
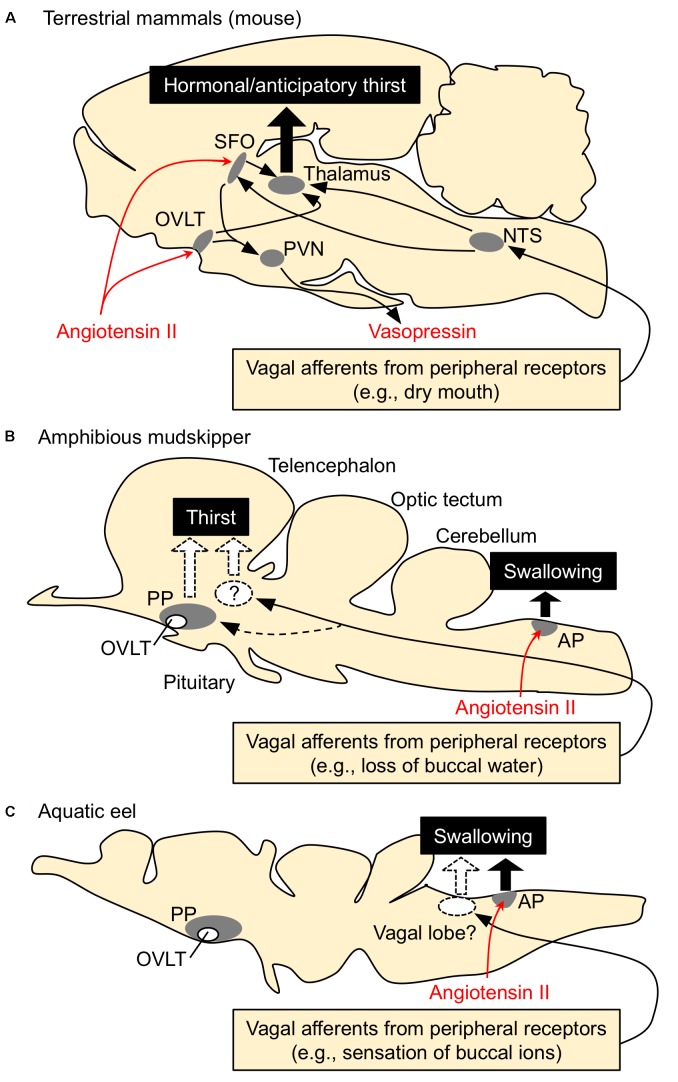
Schematic drawing for the regulatory mechanisms of drinking behavior in terrestrial mammals, amphibious mudskippers, and aquatic eels. Systemic angiotensin II acts on the circumventricular organs (CVOs) that are outside the blood-brain barrier. Among CVOs, the area postrema (AP) and the organum vasculosum of the lamina terminalis (OVLT) exist in mice, mudskippers, and eels, but the subfornical organ (SFO) is identified only in tetrapods. **(A)** Thirst-inducing mechanisms in mice. Systemic angiotensin II is perceived by the neurons in the SFO and OVLT. The signal is transmitted to the thalamus for thirst inducement, and to the paraventricular (PVN) and supraoptic (SON) nuclei for vasopressin secretion. The generation of thirst seems to involve activation of the cortex, which might be mediated by relay neurons in the medial parts of the thalamus. Signals from peripheral receptors (e.g., dry mouth, buccal food) also reach thirst-regulating regions (e.g., SFO) via visceral afferents that course through spinal or vagal pathways. This thirst is evoked before any changes in blood parameters and thus noted as “anticipatory thirst”. NTS, nucleus tractus solitarius. **(B,C)** Mechanisms of drinking in mudskippers and eels. The AP neurons receive systemic angiotensin II and induce swallowing, possibly through the glossopharyngeal-vagal motor complex in the medulla oblongata. In mudskippers **(B)**, sensory detection of loss of buccal water motivates mudskippers to refill water possibly through the vagal afferents, suggesting generation of thirst. Possible thirst center, which regulates migration to water for drinking, has not been identified yet, but vasotocin neurons in the parvocellular preoptic nucleus (PP) might be involved in the neural basis. In eels **(C)**, sensory detection of an increase in Cl^-^ concentration in buccal water induces swallowing of water as an anticipatory drinking. This local stimulus is sensed by afferent fibers of vagus and glossopharyngeal nerves, while the forebrain and AP are not involved in the anticipatory drinking. Regulation of drinking behavior by the vagal afferents appears to be conserved among vertebrates. In contrast, an involvement of the forebrain in the eel drinking has not been implicated.

Thirst is defined as a conscious sensation of a need for water and a desire to drink (Fitzsimons, 1979). In terrestrial animals such as mammals, thirst is followed by a search for water, and its motivation or consciousness is generated in the hypothalamic area and the medial thalamic-cortex network (Denton et al., 1999; Gizowski and Bourque, 2018). Thirst is induced by an increase in systemic Ang II concentration (Kobayashi et al., 1979; Fitzsimons, 1998; Takei, 2000; McKinley and Johnson, 2004). In mammals and birds, systemic Ang II binds to the sensory circumventricular organs (CVOs) in the forebrain that lack the blood-brain barrier to induce drinking behaviors (Simpson and Routtenberg, 1973; Kobayashi and Takei, 1996) (**Figure 2A**). Angiotensin type 1 receptors (AT1) are present in high density in the organum vasculosum of the lamina terminalis (OVLT) and the subfornical organ (SFO) which are known as forebrain CVOs for Ang II-induced thirst (Johnson and Buggy, 1978; Fitzsimons, 1998; McKinley, 2003). Although Ang II also binds to type 2 receptors (AT2), AT2 receptors are sparse at these regions (Rowe et al., 1992). AT1 antagonist losartan inhibited Ang II-induced drinking, but AT2 receptor antagonist PD-123177 did not have any inhibitory action (Timmermans et al., 1993; Goodfriend et al., 1996). Thus, Ang II-induced drinking behavior is mediated through AT1 receptors (Fitzsimons, 1998). It is believed that amphibians such as terrestrial toads and arboreal frogs do not normally drink but instead obtain water by absorption through the ventral skin (Jørgensen, 1997; Bentley, 2002). Interestingly, Ang II induces such water-acquiring behavior called “cutaneous drinking”, in which the pelvic patch is pressed against a moist surface (Hoff and Hillyard, 1991; Propper et al., 1995; Maejima et al., 2010). The cutaneous drinking behavior seems to be regulated via AT1 in the forebrain where CVOs probably localize (Duvernoy and Risold, 2007; Maejima et al., 2010; Uchiyama, 2015), suggesting conserved neural basis of thirst throughout tetrapods. In aquatic teleost fishes, however, none of the regions in the forebrain appear to be involved in elicitation of drinking, since removal of the whole forebrain in eels did not affect the drinking induced by seawater exposure (Hirano et al., 1972) or by injection of Ang II (Takei et al., 1979). These stimuli may act on the hindbrain to initiate swallowing reflex in aquatic teleosts, which complete drinking only by swallowing of buccal water without a search for water (**Figure 2C**). The area postrema (AP) in the hindbrain is proposed to be the primary site of systemic Ang II action, since Evans blue injected into the blood stained this hindbrain CVO (Mukuda et al., 2005) and lesioning of the AP impaired Ang II-induced drinking in eels (Nobata et al., 2013). The AP neurons send cholinergic fibers to the glossopharyngeal-vagal motor complex (Ito et al., 2006), which in turn control the upper esophageal sphincter (UES) muscle (Mukuda and Ando, 2003; Nobata et al., 2013). The UES muscle is the first gate of the alimentary tract and its relaxation leads to initiation of swallowing.

From the comparative point of view, it is intriguing to examine whether amphibious mudskippers have the mechanism inducing thirst as a motivation for drinking. Our recent study showed that peripheral or central administration of Ang II motivates the fish to move to water and to increase the volume of water ingested (Katayama et al., 2018). An OVLT-like structure has been found histologically in the parvocellular preoptic nucleus (PP) of the mudskipper (Hamasaki et al., 2016), but our histochemical study did not support the direct action of Ang II on this region. AT1 receptors have been cloned in teleosts including mudskippers (Nishimura, 2017) although no expression study has demonstrated a calcium signal in the recombinant receptors (Russell et al., 2001). AT1-like mRNA was not detected in the OVLT-like region of mudskippers, while many nuclei in the AP expressed the mRNA (Katayama et al., 2018). AT2 receptors have been cloned in teleosts (Nishimura, 2017), but AT2 mRNA was not detected in the eel brain (Wong and Takei, 2013). Thus, AT1, not AT2, appears to mediate Ang II-induced drinking behavior also in teleosts. In mudskippers, Ang II, through its action on the AP, induced swallowing of buccal water, which is stored on land, and the loss of buccal water motivated mudskippers to move to water (**Figure 2B**; Katayama et al., 2018). Although regulation of the renin-angiotensin system has not been examined when mudskippers are on land, Ang II appears to induce drinking naturally in teleosts since an inhibitor of the renin-angiotensin system (captopril) attenuates spontaneous drinking (Okawara et al., 1987; Perrott et al., 1992; Kobayashi and Takei, 1996). In addition to the osmoregulatory problem, the effect of gravity cannot be nullified in terrestrial environments. Blood pressure of mudskippers is maintained during the transition from submersion to emersion unlike other teleost species, in spite of the influence of gravity (Ishimatsu et al., 1999). Since Ang II contributes to the maintenance of cardiovascular homeostasis in teleosts (Mével et al., 2012; Nishimura, 2017), its relative importance may have been enhanced for cardiovascular regulation as well as for osmoregulation in amphibious mudskippers. In a series of their drinking behaviors, the migration to water stimulated by loss of buccal water is equivalent to the drinking behavior in tetrapods evoked by local stimuli (e.g., dry mouth). Such drinking has been revealed as “anticipatory thirst” in mice because it operates before blood osmolality fluctuates (Berridge, 2004; Zimmerman et al., 2016). In mudskippers, the buccal cavity is filled with water before they exit to land and this behavior appears not to be involved in blood osmolality and hormones. Therefore, the thirst by local sensation may contribute to an anticipatory mechanism to prevent potential dehydration on land.

Although migratory behavior induced by local sensation has not been demonstrated in aquatic fishes, it is well recognized that eels detect an increase in Cl^-^ concentration in buccal water, which enhances swallowing of water (Hirano, 1974). This local stimulus is sensed by afferent fibers of vagus and/or glossopharyngeal nerves (Mayer-Gostan and Hirano, 1976), whereas the forebrain and AP appear not to be involved in the sensory detection because the lesioning of these regions did not attenuate swallowing induced by seawater exposure in the eel (**Figure 2C**; Hirano et al., 1972; Nobata and Takei, 2011). This “chloride response” could prevent future dehydration in hyperosmotic marine environments, and thus was referred to as an anticipatory drinking (Hirano, 1974). The sensation of ions in the buccal cavity of aquatic fishes may be similar to the sensation of buccal water underlying the thirst of tetrapods and mudskippers. In basal vertebrates such as river lamprey (*Lampetra fluviatilis*), the transfer from seawater to fresh water rapidly decreased drinking rate without a change in plasma osmolality (Rankin, 2002). Thus, the mechanism of anticipatory drinking by local sensation appears to be widely distributed among vertebrates. Mudskippers, which evolved from the aquatic teleosts to invade the terrestrial environment (You et al., 2014; Ord and Cooke, 2016), have developed the thirst-inducing mechanism by local sensation, in addition to the hormonal/anticipatory regulation of swallowing at the medulla oblongata observed in totally aquatic fishes (**Figures 2B,C**). Phylogenetically distant vertebrates (ray-finned fish and tetrapods) appear to have acquired the thirst sensation that elicits a series of drinking behaviors when they are exposed to a desiccating environment (Katayama et al., 2018). From the evolutionary point of view, it is intriguing to examine possible thirst mechanisms of amphibious lungfish, which belong to the class Sarcopterygii and are recognized as the closest living relatives of tetrapods (Brinkmann et al., 2004). Since ancestral vertebrates should not have experienced terrestrial environments, thirst may have evolved multiple times during the course of terrestrialization in vertebrates.

In mammals, the input signal for anticipatory thirst was shown to be relayed to the SFO neurons that monitor blood factors such as Ang II (**Figure 2A**; Zimmerman et al., 2016). The SFO orchestrates a motivation for drinking by engaging the medial thalamic-cortex network (Gizowski and Bourque, 2018). Given the complex mechanisms of thirst in mammals, the mudskipper with a simpler brain architecture might be a useful model to investigate the mechanisms of anticipatory thirst by local sensation. In addition to the osmoregulatory purpose, the thirst response also prevents the gills from desiccation, and maintains the branchial respiration in mudskippers (Tamura et al., 1976; Sayer, 2005). Thus, maintaining the moistness of the gill could be one of the selection pressures for the development of thirst. Buccal water is also used for sucking of food when mudskippers eat on land (Michel et al., 2015), and thus water in the cavity decreases after feeding. Because the protrusion and retraction of this water mass is essential for intra-oral transport of prey on land, eating appears to be a potent stimulus for thirst development by local sensation. Many mammalian species drink primarily during meals (Fitzsimons and Le Magnen, 1969; Oatley and Toates, 1969; Berridge, 2004). Food consumption rapidly activated SFO neurons in mammals, beginning at the onset of feeding before any changes in blood parameters occurred (Zimmerman et al., 2016). Activation of SFO neurons during eating was unaffected by angiotensin blockers. Thus, sensory detection of buccal food and its consequent activation of SFO neurons through an angiotensin-independent pathway are indicated for prandial thirst in mammals. In teleosts, however, it has not been examined whether prandial drinking functions in anticipatory fashion to prevent food ingestion-dependent alterations in blood composition. More fish studies on anticipatory drinking, as well as on fast-acting Ang II actions, will be required to know the comprehensive mechanisms for “fast” adaptive response to hyperosmotic environments. Given that anticipatory drinking triggered by local sensation is conserved among vertebrates, comparison of drinking behavior in mudskippers with anticipatory thirst in mammals might provide an answer to the question of why the anticipatory response evolved (Krashes, 2016).

### Neurohypophysial Hormones for Osmoregulation and Social Behaviors

The neurohypophysial hormones, VP and oxytocin (OXT), regulate fluid homeostasis, which requires a tight control of both NaCl and water in mammals (Johnson and Thunhorst, 1997; McKinley et al., 2004). Particularly, antidiuretic VP is a fast- and short-acting hormone that is indispensable for fluid retention in terrestrial environments. VP neurons in the paraventricular (PVN) and supraoptic (SON) nuclei of the hypothalamus react to increases in plasma osmolality by releasing the antidiuretic hormone (Nielsen et al., 1995; Bourque, 2008; Sands et al., 2011; Watts, 2015). The VP neurons are downstream targets of the angiotensinergic neurons innervating the SFO and OVLT (**Figure 2A**; Ferguson, 2009). Systemic Ang II enhances secretion of VP into the circulation through these neural pathways (Zimmerman et al., 2017). Thus, the Ang II-VP system enhances water retention by the kidney. In contrast, OXT decreases ingestive behaviors, including drinking, salt intake, and feeding (Arletti et al., 1990; Blevins et al., 2003; Stricker and Stricker, 2011; Ryan et al., 2017), and increases renal NaCl excretion after a salt load (Balment et al., 1980; Verbalis et al., 1991; Conrad et al., 1993). In addition to systemic osmoregulation, it has recently been suggested that VP neurons and OXT-receptor-expressing neurons anticipate future osmotic fluctuation by drinking, cues predicting water (e.g., visual cue), feeding, or sleeping (Gizowski et al., 2016; Mandelblat-Cerf et al., 2017; Ryan et al., 2017). For example, the neural activity of VP neurons in the PVN and SON, and thereby VP secretion rapidly fell during drinking before any change in blood parameters occurred (Stricker and Stricker, 2011; Mandelblat-Cerf et al., 2017).

In teleosts, neurohypophysial hormones serve for adaptation to a desiccating seawater environment. Transfer of trout to seawater downregulated transcription of VT (the teleost homolog of VP) in the magnocellular preoptic nucleus (PM) of trout (Hyodo and Urano, 1991). In flounders, however, transfer from seawater to fresh water decreased plasma VT concentration (Bond et al., 2002), whereas transfer from fresh water to seawater increased plasma VT levels and VT mRNAs in the hypothalamus (Balment et al., 2006). Thus, VT responses to environmental osmotic challenges and its physiological functions appear to differ among species. IT (the teleost homolog of OXT), as well as VT, has important functions in teleost osmoregulation. VT and/or IT regulate secretion of extra univalent ions in the gill and opercular epithelium (Guibbolini and Avella, 2003; Martos-Sitcha et al., 2015b). These hormones also regulate water transport via aquaporin-1 paralogs, which contribute to water absorption in the intestine for seawater adaptation (Martos-Sitcha et al., 2015a). IT mRNA levels in the hypothalamus increased after transfer to hypersaline media but not to hyposaline media (Martos-Sitcha et al., 2014). These results suggest that IT and its receptor are important for seawater adaptation. In addition to these osmoregulatory functions, VT and IT neurons localized throughout the hypothalamic regions project not only into the pituitary but also into multiple extra-hypothalamic regions, and are known to mediate social behaviors (Holmqvist and Ekstrom, 1995; Thompson and Walton, 2004; Goodson, 2005; Godwin and Thompson, 2012; Lindeyer et al., 2015). Even in mammals, however, little is known about a possible link between osmoregulation and social behaviors, both of which are controlled by the neurohypophysial hormones.

The amphibious behavior in mudskippers may reflect many functions of neurohypophysial hormones that bridge osmoregulation and social behaviors. Mudskippers moved to water when treated with VT or IT either peripherally or centrally (Sakamoto et al., 2015). Migration to water induced by both VT and IT was inhibited by the OXT receptor blocker (H-9405), which specifically induces IT-receptor blockade in teleosts (Watanabe et al., 2007). Expression studies of VT type 1a receptor (V1a) and IT receptors of teleosts in mammalian cell lines indicate that V1a is nearly specific to VT, whereas the sensitivity of the IT receptor to IT is 3-10 times higher than that to VT (Mahlmann et al., 1994; Hausmann et al., 1995; Warne, 2001; Yamaguchi et al., 2012). Thus, neurons expressing IT receptors may regulate amphibious behavior for osmoregulation. However, other VT/IT receptors, especially VT type 2 receptor (V2), might be implicated in the aquatic preference. The V2-type receptor, which is localized in the hypothalamus and osmoregulatory organs, is involved in body fluid homeostasis in teleosts (Konno et al., 2010a,b; Lema, 2010; Martos-Sitcha et al., 2014). When VT or IT was intracerebroventricularly injected, the drinking rate of mudskippers was enhanced by VT, but not by IT (Katayama et al., 2018). Considering the affinities of VT/IT to their receptors, the VT-specific regulation of drinking appears to be mediated by VT receptors in the mudskipper brain. In mammals, VP neurons that innervate the OVLT play an important role in the above-mentioned “anticipatory thirst” (Gizowski et al., 2016). In mudskippers, immunoreactive VT fibers were found in the PP including the OVLT-like region (Hamasaki et al., 2016). These findings suggest that VT may transmit the signal to the neural pathway of thirst-motivated behavior (**Figure 2B**; Katayama et al., 2018). In eels, however, peripherally injected VT reduced drinking rate, but IT increased it (Ando et al., 2000; Nobata and Ando, 2013). Thus, the role of neurohypophysial hormones in regulation of drinking has not been established in teleosts. When mudskippers were dehydrated under terrestrial condition, brain mRNA levels of pro-VT markedly increased while a moderate increase was seen in pro-IT mRNA levels (**Figure 1B**). Given the relatively wide distribution of VT-positive fibers throughout the brain, increased VT under terrestrial condition may naturally stimulate drinking and migration to water (Sakamoto et al., 2015). Nuclei involved in the amphibious behavior were not identified, but brain regions where both VT and IT fibers are localized (e.g., the tuberal nuclei of the hypothalamus, medulla oblongata) may include nuclei expressing IT receptors to regulate this behavior.

**FIGURE 3:**
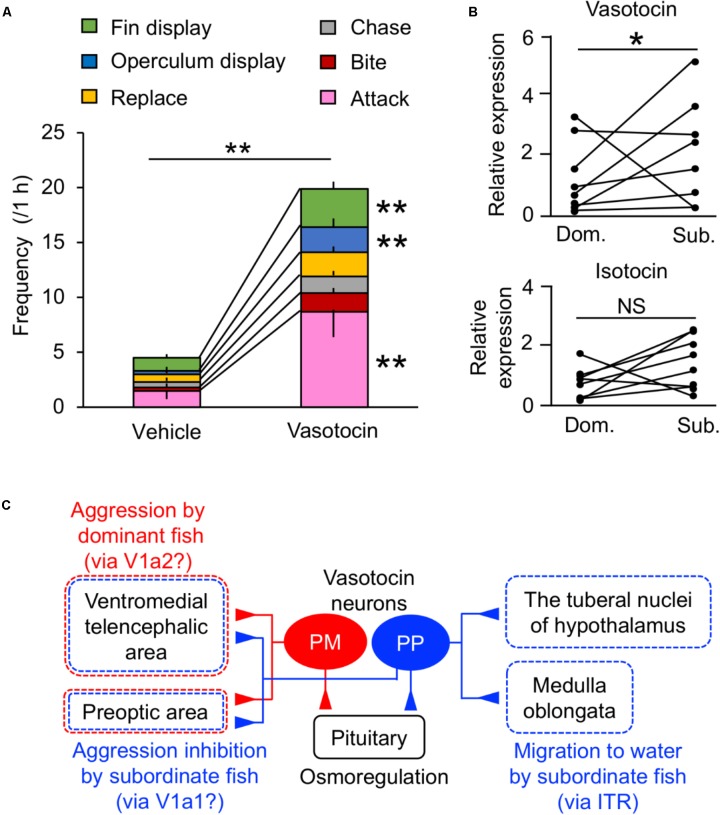
Behavioral actions of vasotocin in mudskippers. **(A)** The frequency of each type of aggressive behavior after injection of vasotocin (500 pg/g-bw) or vehicle in mudskippers (*n* = 6). A size-matched pair of males was used for behavioral observation in a tank with aquatic and terrestrial areas. Data are shown as the means ± SE. ^∗∗^*p* < 0.005 with *t*-test. **(B)** The expression of vasotocin and isotocin mRNAs in dominant (Dom.) and subordinate (Sub.) mudskippers (*n* = 7). Upon introduction in an experimental tank with aquatic and terrestrial areas, a pair of males can be classified as aggressive dominant or submissive subordinate based on the frequency of their aggressive behaviors, which is significantly higher in dominant male. Points of each pair are connected. ^∗^*p* < 0.05 with Mann–Whitney *U*-test. NS, not significant. The original data for **(A)** and **(B)** are published in Kagawa et al. (2013). **(C)** A model illustrating the potential influence of vasotocin neurons on the regulation of aggressive behavior. The cell bodies of vasotocin are localized in the magnocellular (PM) and the parvocellular (PP) preoptic nucleus. Vasotocin may act via V1a-type receptors (V1a1/V1a2) in the ventromedial telencephalic area and the preoptic area to regulate general aggressive behavior. PM cells and V1a2 may mediate aggression by dominant males, while PP cells and V1a1 may mediate submissive behaviors by subordinate males. Since stimulation of migration to water by both vasotocin and isotocin is inhibited by the blocker of isotocin receptor (ITR), brain regions where both vasotocin and isotocin fibers are localized, such as the tuberal nuclei of the hypothalamus and the medulla oblongata, may regulate the amphibious behavior via ITR. The main receptors for specific behaviors are given in parentheses. Broken lines show possible brain regions involved in each behavior.

In addition to the osmoregulatory function, regulation of social behavior by the VT system has been extensively studied in teleosts (Huffman et al., 2015; Yokoi et al., 2015; Loveland and Fernald, 2017; Perrone and Silva, 2018). Central administration of VT in some species indicates that VT neurons mediate aggression, although the directionality (stimulation/inhibition) varies across species (Godwin and Thompson, 2012; Kagawa et al., 2013). In mudskippers, VT specifically regulated general aggressive behavior and/or social communication (i.e., fin display, operculum display, replacement, attacking, chasing, and biting) (**Figure 3A**). In particular, VT-injected males showed significantly higher frequencies of fin display, operculum display and attack than vehicle-injected males. The former two types of behaviors are less aggressive than the latter one, and it is suggested that the VT might modulate social communication as well as aggression in mudskippers. Pro-VT mRNA levels in the whole brain of subordinate, however, were higher than in that of dominant (**Figure 3B**). In several freshwater teleosts, aggressive dominant males have high VT expression in the PM, whereas the submissive subordinate males have high VT expression in the PP (Larson et al., 2006; Greenwood et al., 2008; Kagawa, 2013). Together with prolonged aquatic stay by VT-injected mudskippers, VT in the PP may play a characteristic role in promoting migration into water for submissive subordinates relative to aggressive dominants (Kagawa et al., 2013). VT expression in the PP is involved in the hormonal stress response in the European eel and the rainbow trout (Olivereau and Olivereau, 1990; Gilchriest et al., 2000). However, the migration into water by VT-injected mudskippers cannot be fully explained by a physiological stress response, since there was no difference in plasma cortisol levels (Kagawa et al., 2013). In the mudskipper brain, VT fibers were localized in the preoptic and ventromedial telencephalic areas (Sakamoto et al., 2015). These regions include V1a-expressing neurons in teleosts (Kline et al., 2011; Huffman et al., 2012; Lema et al., 2015). With regard to V1a receptor subtypes, telencephalic V1a1 levels were higher in subordinates compared to dominants, and levels of V1a2 in the telencephalon of dominant males correlated with aggression in killifish (Lema et al., 2015). In cichlid fish, the ventromedial telencephalic area was the site of high density expression for both of these receptors (Loveland and Fernald, 2017). These findings suggest that VT neurons projecting to the ventromedial telencephalic area and/or preoptic area act via V1a2 in the dominant mudskipper to elicit general aggressive behavior, and that the VT neurons act via V1a1 in the subordinate to inhibit general aggressive behavior (**Figure 3C**). As described above, VT neurons projecting to the tuberal nuclei of the hypothalamus and medulla oblongata may act at least in part via IT receptors to stay in the aquatic habitat forced by the dominant fish (**Figure 3C**). Given the suite of processes mediated by neurohypophysial hormones, migration of subordinate mudskippers into water reflects a unique interaction between the hormonal regulations of social and osmoregulatory behaviors from competitive and dehydrating land to aquatic environments in the amphibious teleost. A few studies on teleosts have shown that IT controls the reproductive behavior and/or spawning act (Gonçalves and Oliveira, 2011). Mudskippers spawn in their mudflat burrows filled with water, and secure embryonic development within the hypoxic burrows by transporting mouthfuls of air (Ishimatsu et al., 2007). Thus, the unique link between osmoregulation and reproduction regulated by IT should be possible.

### Corticosteroids for Ion Regulation and Stress Response

**FIGURE 4:**
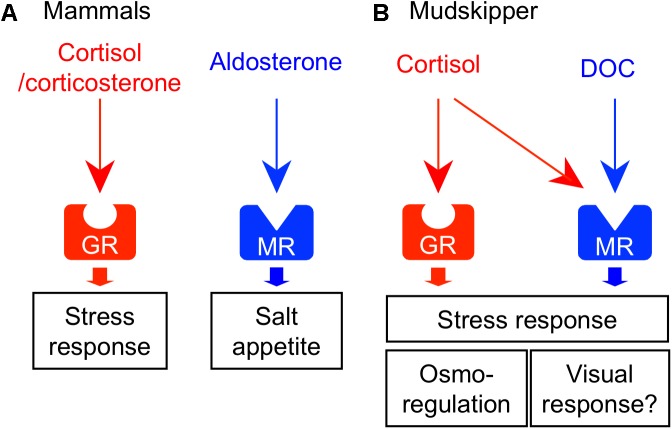
Receptor-ligand interactions of corticosteroids and their central actions. **(A)** Corticosteroid system in mammals. Glucocorticoid (cortisol/corticosterone) and mineralocorticoid (aldosterone) activate their respective receptors – glucocorticoid receptor (GR) and mineralocorticoid receptor (MR). These corticosteroids act on the various brain regions and regulate homeostatic behaviors such as stress response and salt appetite. **(B)** Corticosteroid system in mudskippers. Cortisol-GR system mediates both glucocorticoid and mineralocorticoid functions. 11-deoxycorticosterone (DOC) is the preferred ligand for MR. In mudskippers, both MR and GR regulate the amphibious behavior related to a stress response. The aquatic preferences induced by GR and MR signaling may be related to osmoregulation and visual response, respectively.

Corticosteroids function as glucocorticoids and mineralocorticoids in vertebrates. Glucocorticoids regulate metabolism and growth, while mineralocorticoids regulate the body fluid osmolality. In tetrapods, these functions are achieved by two distinct hormones: cortisol/corticosterone (glucocorticoids) and aldosterone (mineralocorticoid). Glucocorticoids and mineralocorticoids activate their receptors – the glucocorticoid receptor (GR) and mineralocorticoid receptor (MR), respectively (**Figure 4A**). In the mammalian brain, the GR and MR are both highly expressed in the hippocampus and in several hypothalamic nuclei such as PVN and arcuate nucleus. Co-localization of these receptors has been found in most neurons of the nuclei. These findings suggest that the expression balance of GR/MR within the nucleus is critical for many physiological and short-term behavioral responses, such as regulation of salt intake, mood, appetite, and exploratory behavior (Rozeboom et al., 2007; Kawata et al., 2008; Geerling and Loewy, 2009; Sakamoto et al., 2012). The CVOs such as the SFO and the AP are considered to be involved in the synergistic action of Ang II and mineralocorticoid on salt appetite (Epstein, 1982; Fluharty and Epstein, 1983; Alhadeff and Betley, 2017), since the neurons in those brain areas express both angiotensin receptor (Gehlert et al., 1991; Tsutsumi and Saavedra, 1991; Song et al., 1992) and MR (Coirini et al., 1983).

As mentioned earlier, cortisol functions not only as glucocorticoid but also as mineralocorticoid in teleosts (Mommsen et al., 1999). Two different GR coding genes (GR1 and GR2) and one MR gene have been found in this fish group (Bury et al., 2003; Greenwood et al., 2003). Cortisol interacts with MR as well as with GRs, but cortisol-MR axis appears not to be important for osmoregulation unlike in mammals (Prunet et al., 2006; Stolte et al., 2008) (**Figure 4B**). Indeed, a constitutive MR-knockout medaka can grow and adapt to seawater, as well as to fresh water (Sakamoto et al., 2016). By contrast, inhibition of the GR by RU-486 prevented killifish from seawater adaptation (Shaw et al., 2007). Many studies using euryhaline teleosts indicate that cortisol-GR system plays important roles in both seawater and freshwater adaptation as a slow-acting hormone (McCormick, 2001; Takahashi and Sakamoto, 2013; Takei et al., 2014). In many teleosts, plasma cortisol and GR transcripts in osmoregulatory organs changed after transfer to seawater or fresh water although the directionality varied across species (McCormick, 2001; Takahashi and Sakamoto, 2013). In mudskippers (Sakamoto et al., 2002), plasma cortisol concentrations markedly increased when the teleosts were dehydrated under terrestrial condition (**Figure 1B**). Cortisol stimulated epithelial apoptosis in the mudskipper esophagus so that NaCl was desalinated from ingested seawater. Cortisol also induced cell proliferation to reduce permeability for freshwater adaptation (Takahashi et al., 2006). The dual functions of cortisol in teleosts may stem from the distinct action on multiple GR isoforms with different sensitivities to cortisol for transactivation and transrepression activities (Prunet et al., 2006; Stolte et al., 2008). In the gills of some teleosts, cortisol stimulated the differentiation of ionocytes into seawater type or freshwater type, and elevated the activity and/or transcription of key transporters in ionocytes such as NKA, Na^+^-K^+^-2Cl^-^ cotransporter type 1, and cystic fibrosis transmembrane conductance regulator (Deane et al., 2000; McCormick, 2001; Aruna et al., 2012; Takahashi and Sakamoto, 2013). These actions increased ion excretion for seawater adaptation and ion uptake for freshwater adaptation, respectively. Cortisol also elevated the NKA activity and aquaporin expression in the intestine, thereby increasing water absorption across the epithelia to maintain water balance in a dehydrating seawater environment (Veillette et al., 1995; Veillette and Young, 2005; Cutler et al., 2007). These studies suggest that the hypo- and hyper-osmoregulatory action of cortisol-GR system is well conserved among euryhaline teleosts (Takei et al., 2014).

In teleosts, the circulation of aldosterone, present in extremely low levels, is unlikely to have actions on the GRs or MRs (Prunet et al., 2006). However, 11-deoxycorticosterone (DOC) is a circulating corticosteroid that is present in significant concentrations, and can activate MRs but not GRs in teleosts (Sturm et al., 2005; Prunet et al., 2006; Milla et al., 2008; Stolte et al., 2008). In expression studies in mammalian cell lines, transactivation of the teleost MR is 10 times more sensitive to DOC than to cortisol, whereas the teleost GR is specific to cortisol (Sturm et al., 2005; Prunet et al., 2006; Stolte et al., 2008). In agreement with the presence of the ligand for MRs in the plasma, the teleost MR mRNA was found in many tissues (Greenwood et al., 2003; Sturm et al., 2005; Arterbery et al., 2010). The expressions of MR mRNA are relatively modest in osmoregulatory organs involved in ionoregulation (e.g., gill), but considerably higher in the brains of most teleosts examined (e.g., Sakamoto et al., 2016). These recent finding suggested that MR system may carry out some behavioral functions in teleosts. In fact, mudskippers migrated into water when treated with DOC and cortisol (Sakamoto et al., 2011). Cortisol may act as an endogenous ligand for the brain MRs to stimulate the migration to water naturally, since plasma cortisol, rather than DOC, increased in mudskippers under terrestrial condition (**Figure 1B**). However, MRs can be insensitive to cortisol activation *in vivo* because 11β-hydroxysteroid dehydrogenase type 2 (11β-HSD2) catalyzes the conversion of cortisol to the MR-inactive cortisone (Funder et al., 1988). Without the expression of 11β-HSD2, MRs probably function as cortisol receptors. Hence, study on localization of 11β-HSD2 in the teleost brain is further required. The aquatic preference in 10 ppt seawater, possibly stimulated by the brain MR signaling, may reflect the induction of salt appetite as shown by aldosterone in mammals (Alhadeff and Betley, 2017). Thus, it is of interest to examine synergistic effects of Ang II and corticosteroids to evaluate salinity preference of mudskippers using an aquarium test system. In contrast, the GR signaling may also contribute to the aquatic preference because the cortisol-stimulated behavior was not completely inhibited by the specific GR blocker, RU-486. Since the cortisol-GR system is implicated in excretion of extra ions by elevating the NKA activity in teleosts (McCormick, 2001; Takahashi and Sakamoto, 2013), mudskippers may migrate to water for ion excretion through the skin under the pectoral fin (Sakamoto et al., 2000, 2002). The distinct function of MR and GR signaling should be investigated in the osmoregulatory behavior of mudskippers.

In addition to the osmoregulatory function, GRs and MRs appear to regulate stress responses in the teleost brain (Takahashi and Sakamoto, 2013; Myers et al., 2014; Sakamoto et al., 2018). In teleosts, GRs and MRs are localized in key components of the stress axis, such as the forebrain pallial area, the corticotrophin-releasing hormone cells in the preoptic nucleus, and the adrenocorticotropic-hormone cells in the pituitary pars distalis (Stolte et al., 2008; Kikuchi et al., 2015; Sakamoto et al., 2016). The aquatic preference of mudskippers, stimulated by the brain MR/GR signaling, may also be a stress response, since the dehydrated mudskipper under terrestrial condition appears to be stressed (**Figure 4**; Sakamoto et al., 2002). Such a system that is responsive to external stressors can also mediate the start of migration from river to ocean in salmon (Clements and Schreck, 2004; Flores et al., 2012). The expression of GRs and MRs mRNA were observed in most of the PM and PP, known to produce VT and IT (Teitsma et al., 1998), and the cortisol-GR system regulated VT and IT release from the hypothalamus-pituitary complex (Kalamarz-Kubiak et al., 2015). Thus, future studies should focus on the “cross-talk” among these hormones in the brain to clarify the link between osmoregulation and stress response, both of which are primarily regulated by the neurohypophysial hormones and corticosteroids. Furthermore, GR mutant adult zebrafish became immobile with reduced exploratory behavior when placed into an unfamiliar aquarium (Ziv et al., 2013). The mutant did not habituate to this stressor upon repeated exposure. Addition of the antidepressant fluoxetine or visual interactions with a wild type fish restored normal behavior. Thus, GR signaling appears to contribute to mood regulation, as well as to the stress response. In contrast, MR-knockout medaka failed to track moving dots although the swimming motility of the mutant increased (Sakamoto et al., 2016). Thus, MR is required for normal activity of locomotion in response to visual motion stimuli. Vision is more important in terrestrial lifestyle than in aquatic one, and sophisticated vision might have promoted land invasion in vertebrates (MacIver et al., 2017). Mudskippers with their unique vision system (Takiyama et al., 2016) will be a good model to analyze the evolution of corticosteroids-regulated vision response.

## Conclusion and Perspectives

**FIGURE 5:**
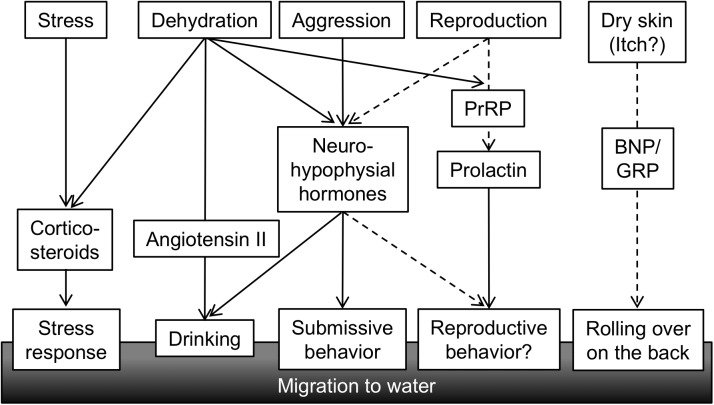
A conceptual diagram of hormones regulating amphibious behavior of mudskippers. Various stimuli related to the amphibious lifestyle of mudskipper activate secretion of hormones, which in turn leads to behavioral and physiological outputs. Solid lines showing functional associations are based on published data, while broken lines are speculation. PrRP, prolactin-releasing peptide; BNP, B-type natriuretic peptide; GRP, gastrin-releasing peptide.

In this review, we summarized the role of Ang II, neurohypophysial hormones, and corticosteroids in the regulation of amphibious behavior in mudskippers. Mudskippers migrate to water for drinking and for escape from dominant conspecifics and stressful situations (**Figure 5**). The analyses of their drinking patterns suggest that the neural basis of amphibious behavior is connected to a water detection system in the buccal cavity, which is related to induction of thirst. Direct action of systemic Ang II on the OVLT-like structure and the Ang-II/neurohypophysial hormone axis in the forebrain have not yet been investigated in teleosts including mudskippers. In future research, the target site(s) of systemic Ang II other than those along the lamina terminalis might be identified in the mudskipper forebrain. VT also regulates amphibious behavior related to aggression/submission as well as to drinking, suggesting their distinct functions in each site of the brain. Cortisol may bind to the MR in the brain to elicit a preference for aquatic habitation, which reveals a conserved central action of mineralocorticoid signaling throughout vertebrates (Sakamoto et al., 2018).

It remains to be discovered how other behaviors regulated by osmoregulatory hormones have evolved during vertebrate terrestrialization, and these research gaps will need to be addressed in future study (**Figure 5**). As described above in regard to IT function, mudskippers spawn in their mudflat burrows that are filled with water, and they secure embryonic development of their young within the hypoxic burrows by transporting mouthfuls of air (Ishimatsu et al., 2007). These unique reproductive behaviors including both migration and parental care might be regulated by osmoregulatory hormones such as IT and prolactin (PRL). PRL plays a critical role in freshwater adaptation in teleost fishes (Manzon, 2002; Sakamoto and McCormick, 2006). PRL reduces ion and water permeability of osmoregulatory surfaces in fresh water, and increases ion uptake (Hirano, 1986; Manzon, 2002; Sakamoto et al., 2005b; Shu et al., 2016). Further, many of the reproductive functions of PRL appear to be conserved throughout the vertebrates (Whittington and Wilson, 2013). Migration plays an important role in the reproductive cycle of many vertebrates. For example, PRL injection induced migration from land to water in salamanders (Moriya, 1982). In the amphibious behavior of mudskippers, the PRL-releasing peptide/PRL axis induced a preference for aquatic habitation (Sakamoto et al., 2005b). This action resembles the migration to water of salamanders for spawning. PRL transcription and secretion were promoted by PRL-releasing peptide (Sakamoto et al., 2003; Fujimoto et al., 2006). In mudskippers, mRNA levels of PRL-releasing peptide and of PRL are similarly regulated. The mRNA levels in the brain-pituitary axis increased during both terrestrial and freshwater acclimation (Sakamoto et al., 2005a), although the dynamics of PRL mRNA during spawning has not been examined in mudskippers. During the chum salmon maturation process, PRL mRNA levels increased with the onset of anadromy (Onuma et al., 2010), whereas PRL may have no role in the reproductive migration of catadromous eels (Sudo et al., 2013). Thus, the role of PRL in reproductive migration appears to depend on environmental conditions where the teleosts live, and may have become important in the amphibious lifestyles of mudskippers. In teleosts, like in mammals, PRL also regulates reproductive development and brood care behavior as well as migration (Whittington and Wilson, 2013). Mudskippers showing such various reproductive traits can be fascinating models to explore hormonal function in behaviors related to both osmoregulation and reproduction.

Furthermore, in mudskippers, aquatic preference and rolling behavior on wet land are notable for moistening the dorsal skin (Ip et al., 1991). Since natriuretic peptides and gastrin-releasing peptide are currently known as key molecules to transmit itch sensation to the central nervous system in rodents (Sun and Chen, 2007; Mishra and Hoon, 2013; Liu et al., 2014), further analyses of these peptides may elucidate relationships between habitats and itch sensation by dry skin, as well as the unknown evolution of the itch sensation in vertebrates. As mentioned already, the “cross-talk” among these hormones in mudskippers may explain the coordination of amphibious behavior and other physiological regulation throughout vertebrate species.

## Author Contributions

All authors listed have made a substantial, direct and intellectual contribution to the work, and approved it for publication.

## Conflict of Interest Statement

The authors declare that the research was conducted in the absence of any commercial or financial relationships that could be construed as a potential conflict of interest.

## Abbreviations

11β-HSD2: 11β-hydroxysteroid dehydrogenase type 2
Ang II: angiotensin II
AP: area postrema
AT1: angiotensin type 1 receptor
AT2: angiotensin type 2 receptor
BNP: B-type natriuretic peptide
CVOs: circumventricular organs
DOC: 11-deoxycorticosterone
GR: glucocorticoid receptor
GRP: gastrin-releasing peptide
IT: isotocin
ITR: isotocin receptor
MR: mineralocorticoid receptor
NKA: Na^+^-K^+^-ATPase
NTS: nucleus tractus solitarius
OVLT: organum vasculosum of the lamina terminalis
OXT: oxytocin
PM: magnocellular preoptic nucleus
PP: parvocellular preoptic nucleus
ppt: parts per thousand
PRL: prolactin
PrRP: prolactin-releasing peptide
PVN: paraventricular nuclei of the hypothalamus
SFO: subfornical organ
SON: supraoptic nuclei of the hypothalamus
UES: upper esophageal sphincter
VP: vasopressin
VT: vasotocin
V1a: vasopressin/vasotocin type 1a receptor
V2: vasopressin/vasotocin type 2 receptor
